# National equity of health resource allocation in China: data from 2009 to 2013

**DOI:** 10.1186/s12939-016-0357-1

**Published:** 2016-04-19

**Authors:** Wen Liu, Ying Liu, Peter Twum, Shixue Li

**Affiliations:** Department of Social Medicine and Health Management, College of Public Health, Shandong University, 44 Culture Road, Li Xia District, Jinan, 250012 Shandong Province China; Department of Epidemiology and Biostatistics, College of Public Health, Shandong University, 44 Culture Road, Li Xia District, Jinan, 250012 Shandong Province China

**Keywords:** Equity, Health care resources, Theil index

## Abstract

**Background:**

The inequitable allocation of health resources is a worldwide problem, and it is also one of the obstacles facing for health services utilization in China. A new round of health care reform which contains the important aspect of improving the equity in health resource allocation was released by Chinese government in 2009. The aim of this study is to understand the changes of equity in health resource allocation from 2009 to 2013, and make a further inquiry of the main factors which influence the equity conditions in China.

**Methods:**

Data resources are the China Health Statistics Yearbook (2014) and the China Statistical Yearbook (2014). Four indicators were chosen to measure the trends in equity of health resource allocation. Data were disaggregated by three geographical regions: west, central, and east. Theil index was used to calculate the degree of unfairness.

**Results:**

The total amount of health care resources in China had been increasing in recent years. However, the per 10, 000 km^2^ number of health resources showed a huge gap in different regions, and per 10, 000 capita health resources ownership showed a relatively small disparities at the same time. The index of health resources showed an overall downward trend, in which health financial investment the most unfair from 2009 to 2012 and the number of health institutions the most unfair in 2013. The equity of health resources allocation in eastern regions was the worst except for the aspect of health technical personnel allocation. The regional contribution rates were lower than that of the inter-regional contribution rates which were all beyond 60 %.

**Conclusion:**

The equity of health resource allocation improved gradually from 2009 to 2013. However, the internal differences within the eastern region still have a huge impact on the overall equity in health resource allocation. The tough issues of inequitable in health resource allocation should be resolved by comprehensive measures from a multidisciplinary perspective.

## Background

Fairness is the common ideal of human society, with the increase of the social wealth level, the degree of attention to fairness issues continues to improve. The equity of the health resource allocation which means the way health resources been distributed and flowed among the health care departments or regions can reflect the degree of health equity which is an important aspect of social fairness [1]. Equitable allocation of health resources is one of the basic conditions to achieve the population health fairness. It is also one of the main goals for governments’ interventions in the health services market. In other words, the government should take measures to intervene the allocation of health resources in order to use fewer resources for more returns of health improvement. The basic ethical principles of health resource allocation advocated by the World Health Organization are equity, efficiency and utility [2]. World Health Organization goal of health policy advocated in recent years is to ensure equitable health service provision through the rational allocation of health resources. They declared that it’s the fundamental right for every human being to enjoy the highest standard of health, irrespective of religion, race, economic/social or political belief. From a practical point of view, equitable distribution of health resources has been regarded as the most important aspect of a full 38 regional health assessment indicators in Europe [3]. Britain and other countries have set up a specialized working group on allocation of health resources with the basic principle of people with the same health needs have the same health care access. The inequity of the health resource allocation is a global problem, not just a common phenomenon in China or other developing countries. In 2009, the Chinese Ministry of Health issued new medical reform program, one of the five reform tasks was to further enhance the national health care system. The developed areas should strengthen their support for the backward areas on equipment, health technical personnel and financial assistance via long-term cooperation and reconstruction work. The provincial government should also improve the regional health planning, which including of medical resources integration and sharing in different regions, at the same time, focusing on the weak links such as rural and community health resources investment. That is to say, it is necessary to optimize the allocation of limited resources to meet the health service demands of the population [4].

The equity of health resource allocation has attracted the interests of many scholars in the field of public health. Researchers focused on the measurement of equity from various aspects. Firstly, several different methods were adopted to conduct the research on equity [5], such as Gini-coefficient and Lorenz curve [6]; Theil index [7]; concentration index [8]; Atkinson index [9] and so on. Each method has its’ own disadvantages and advantages. Lorenz curve could put a vivid reflection of the equity in resources allocation when combine with Gini coefficients. It can also compare the different situations over time and between geographic areas. But Gini coefficients are more sensitive in the upper classes and can only reflect the overall degree of differences. Concentration index which is sensitive to different social classes can take the socioeconomic factors into consideration when calculate the indexes of equity. It can reflect the overall inequity but not containing the variables of resource delivery itself. Atkinson index is done by setting an external display of regional differences in parameters related to the evaluation, and the higher the parameter settings, the larger of the regional differences. It is suitable to analyze the small differences particularly. When compared with other methods, Theil index is more sensitive to the efficiency of resource allocation. It can reflect the contribution rate within the group and between groups when measuring the main factors causing the disparities. Secondly, most researchers explored the equity from only one aspect of health resources allocation or the viewpoint of a single province, such as the health technical personnel resources, health input. Some researchers explored from the viewpoint of health resources distribution which are related to the particular diseases [10]. Thirdly, most of the researches were based on the cross section methods, which had no thorough discussions to the develop trend of equity. In this study, we used the method of Theil index to explore the changes of equity in health resource allocation over time. We also discussed the main factors influencing the equity in different regions.

## Methods

### Data resources and regional division

In this study, we obtained the relevant data from the China Health Statistics Yearbook 2014 and China Statistical Yearbook 2014. We studied all 31 provinces, autonomies and municipalities of mainland China. Due to the inconsistency of statistical standards, the data did not include Macao and Hong Kong Special Administrative Regions and province of Taiwan. According to the geographical position and the level of the Gross Domestic Product (GDP) per capita, all the 31 provinces were divided into three groups: the backward western region, the developing middle region and the most developed eastern region. Inner Mongolia, Guangxi, Chongqing, Sichuan, Guizhou, Yunnan, Tibet, Shanxi, Gansu, Qinghai, Ningxia, Xinjiang belong to the western region. Shanxi, Jilin, Heilongjiang, Anhui, Jiangxi, Henan, Hubei, Hunan belong to the middle region. The eastern region including Beijing, Tianjin, Hebei, Liaoning, Shanghai, Jiangsu, Zhejiang, Shandong, Guangdong, and Hainan.

### Measuring tool

Theil index was first used by a famous economist Theil (1967) who explored the entropy theory to evaluate the equity of income [11]. The Theil index ranges from 0 to 1, the smaller the value, the more equitable the different regions will be. Theil index was originally used as a measuring tool of income equity, but an increasing number of experts in public health tried to adopt this method to explore the equity of health care and health resource allocation. The formula of the Theil index is shown as follows;

1 $$ T={\displaystyle \sum_{i=1}^n{p}_i \ln \frac{p_i}{y_i}} $$

In the above formula (3), *p*_i_ represents the population proportion accounts for total population in a region; *y*_i_ represents the health resources accounts for the total number of health resources in a region. The total Theil index could be divided into two groups, which called the “within group” and the “between groups”. The decomposition formula of Theil index is as follows;

2 $$ {T}_{\operatorname{int}ra}={\displaystyle \sum_{g=1}^k{p}_g{t}_g} $$

3 $$ {T}_{\operatorname{int}er}={\displaystyle \sum_{g=1}^k{p}_g \ln \frac{p_g}{y_g}} $$

4 $$ T={T}_{\operatorname{int}ra}+{T}_{\operatorname{int}er} $$

*T*_intra_ represents the degree of health resources allocation fairness in the area; *T*_inter_ represents the degree of health resources allocation fairness between the different areas; *p*_g_ and *y*_g_ means the same with *p*_i_ and *y*_i_. The contribution of “within group” and the “between groups” can be calculated by dividing *T* [12].

### Main indicators

The Theil Index was estimated with 4 indicators which including the number of health care institutions, the number of beds in health care institutions, the number of health technical personnel and the health investment amount. We analyze the allocation of health resources from the perspective of population distribution.

Specifically, inclusion and exclusion criteria of the four indicators are shown as following.

1. Health care institutions refers to the institutions such as hospitals, primary health care institutions, professional public health agencies and other health care institutions which have obtained the legal registration certificates from the health administrative departments.
2. Beds in health care institutions refer to the actual number of beds in medical institutions, including formal beds, simple beds, care beds, beds are being disinfected or repaired, not including neonatal beds, pre-delivery beds, observation beds, temporary beds and the accompany beds for patients' family.
3. Health technical personnel refers to the practicing physicians, practicing physician assistants, registered nurses, pharmacists, radiologists, health supervisors and trainee physicians and other health professionals which not including those engaged in the management of health workers, such as president, vice president, party secretary.
4. Health investment refers to all levels of government funding for health services, health care subsidies, health care administration, population and family planning affairs expenditures and other undertakings.

## Result

### Time trend in health care resources of China from 2009 to 2013

Changes in the amount of health care resources were shown in Table 1. Totally, the health care resources in China had been increasing in recent years. The basic figure of population had been grown from 13.3 billion to 13.6 billion, and the per capita health care resources of four kinds were all increased at the same time. The amount of health investment which made the largest increase in 2013 was twice than that in 2009. Per 10,000 capita number of beds, technical personnel and health care institutions rose 37.3, 25.8, 4.22 % respectively.

**Table 1:** Basic information on health resource allocation from 2009 to 2013

Year		Health care institutions		Health technical personnel		Health care beds		Health investment	
	Population (10,000 persons)	Number (unit)	Per 10,000 persons	Number (individuals)	Per 10,000 persons	Number (unit)	Per 10,000 persons	Amount (hundred million yuan)	Per 10,000 persons
2009	133450	916571	6.87	5535124	41.48	4416612	33.10	3930.69	0.03
2010	134091	936927	6.99	5866158	43.75	4786831	35.70	4730.62	0.04
2011	134735	954389	7.08	6192858	45.96	5159889	38.30	6358.20	0.05
2012	135404	950297	7.02	6668549	49.25	5724775	42.28	7170.81	0.05
2013	136072	974398	7.16	7200578	52.92	6181891	45.43	8203.20	0.06

### Regional distribution of health resources in 2013

In order to make a deeper understanding of the resources distribution in China, we made a calculation of the per 10, 000 capita health care resources in the year of 2013. For better comparison, per 10, 000 km^2^ resources distribution were also listed. The specific values were shown in Tables 2 and 3.

**Table 2:** Regional distribution of per capita health resources in 2013

Region	Population (10,000 person)	Health care institutions	Health technical personnel	Beds in health care institutions	Health investment
Shanghai	2415	2.04	65.05	47.33	0.09
Tianjin	1472	3.19	55.08	39.19	0.09
Jiangsu	7939	3.90	54.02	46.39	0.06
Anhui	6030	4.09	42.05	39.14	0.06
Guangdong	10644	4.49	52.02	35.55	0.05
Beijing	2115	4.58	96.34	49.18	0.13
Yunnan	4687	5.18	41.23	44.83	0.06
Zhejiang	5498	5.47	64.11	41.85	0.06
Hei Longjiang	3835	5.57	54.13	49.33	0.05
Hainan	895	5.60	53.74	35.85	0.08
Hubei	5799	6.14	53.34	49.70	0.06
Chongqing	2970	6.37	47.86	49.63	0.07
Ningxia	654	6.47	57.00	47.54	0.08
Guangxi	4719	7.19	51.05	39.67	0.06
Jilin	2751	7.24	53.04	48.41	0.07
Fujian	3774	7.47	52.34	41.36	0.06
Henan	9413	7.59	49.77	45.66	0.05
Shandong	9733	7.75	61.33	50.31	0.05
Liaoning	4390	8.11	58.02	55.10	0.05
Xinjiang	2264	8.24	64.41	60.64	0.07
Guizhou	3502	8.33	44.52	47.60	0.07
Jiangxi	4522	8.60	42.04	38.54	0.06
Hunan	6691	9.30	48.29	46.95	0.05
Inner Mongolia	2498	9.31	59.34	48.09	0.08
Sichuan	8107	9.87	52.67	52.62	0.06
Shanxi	3764	9.87	63.51	49.18	0.07
Gansu	2582	10.34	45.73	44.96	0.06
Qinghai	578	10.42	56.13	51.06	0.12
Hebei	7333	10.70	45.42	41.39	0.05
Shanxi	3630	11.10	56.03	47.55	0.06
Tibet	312	21.55	37.30	35.25	0.13

**Table 3:** Regional distribution of per 10, 000 km^2^ health resources in 2013

Region	Area (10,000 square kilometers)	Health care institutions	Health technical personnel	Beds in health care institutions	Health investment
Shanghai	0.63	0.78	24.94	18.14	341.14
Tianjin	1.13	0.41	7.18	5.11	114.11
Jiangsu	10.26	0.30	4.18	3.59	46.38
Anhui	13.97	0.18	1.81	1.69	25.90
Guangdong	18	0.27	3.08	2.10	31.63
Beijing	1.68	0.58	12.13	6.19	164.36
Yunnan	38.33	0.06	0.50	0.55	7.84
Zhejiang	10.2	0.29	3.46	2.26	34.39
Hei Longjiang	45.48	0.05	0.46	0.42	4.19
Hainan	3.40	0.15	1.41	0.94	20.47
Hubei	18.59	0.19	1.66	1.55	17.33
Chongqing	8.23	0.23	1.73	1.79	24.06
Ningxia	6.64	0.06	0.56	0.47	8.10
Guangxi	23.6	0.14	1.02	0.79	12.10
Jilin	18.74	0.11	0.78	0.71	9.69
Fujian	12.13	0.23	1.63	1.29	18.49
Henan	16.7	0.43	2.81	2.57	29.49
Shandong	15.38	0.49	3.88	3.18	31.59
Liaoning	14.59	0.24	1.75	1.66	15.73
Xinjiang	166.00	0.01	0.09	0.08	0.97
Guizhou	17.60	0.17	0.89	0.95	12.99
Jiangxi	16.70	0.23	1.14	1.04	15.70
Hunan	21.18	0.29	1.53	1.48	16.17
Inner Mongolia	118.3	0.02	0.13	0.1	1.66
Sichuan	48.14	0.17	0.89	0.89	10.12
Shaanxi	20.56	0.18	1.16	0.9	12.51
Gansu	45.44	0.06	0.26	0.26	3.65
Qinghai	72.23	0.01	0.04	0.04	0.95
Hebei	18.77	0.42	1.77	1.62	20.28
Shanxi	15.63	0.26	1.30	1.10	12.90
Tibet	122.80	0.01	0.01	0.01	0.33

From the perspective of per 10, 000 capita, we can get an initially understand of resources distribution in the population from different areas. In terms of the health care institutions, most of the values were concentrated in 5.5 to 10. But we can also find the extreme differences between the two ends of the values. The number of health care institutions per 10, 000 people in Tibet (21.55) was 10 times more than that in Shanghai (2.04) while the western regions 3 times more than that in the eastern. Compared to the number of health care institutions, the degrees of discrepancy in other 3 resources were not as great. Per 10, 000 capita of health investment among the 31 areas showed the smallest gap.

The comparative analysis of geographical distribution of health care resources showed a completely different situation. In all the 31 regions, Shanghai ranked first whether we focused on the perspective of health care institutions’ number per 10, 000 km^2^ or the other 3 types resources, while Beijing got the second place. However, the number of health technical personnel per 10, 000 km^2^ showed us a shocking disparity which was even more than 2494 times between the top one and the last one in the rankings.

### Theil index of health care resources allocation based on population

The results shown in Tables 2 and 3 present the situation of health resource allocation in the level of province or municipalities, from which we found a large gap among the regions. Overall, the share of health resources in the eastern region is higher than that in the other regions, while the western has the lowest rate of health resources possession. To further verify, comparative analysis of the Theil Index were made in the three regions from 2009 to 2013. Theil index of health resources allocation is shown in Table 4.

**Table 4:** Theil index of health resources allocation from 2009 to 2013

Year	Health care institutions	Health technical personnel	Beds in health care institutions	Health investment
2009	0.050	0.011	0.004	0.123
2010	0.055	0.011	0.005	0.128
2011	0.057	0.009	0.004	0.124
2012	0.057	0.009	0.003	0.126
2013	0.057	0.007	0.003	0.011

From the year of 2009 to 2012, the Theil index of health investment was significantly higher than other kind of resources. While in 2013, it dropped from about 0.12 to 0.01, which was still higher than that of health technical personnel (0.007) and beds in health care institutions (0.003), but lower than the Theil index of health care institutions (0.057). The Theil index of health care institutions was about 0.05 or a bit more. The index of the other two health resources showed an overall downward trend in these years.

**Fig. 1:**
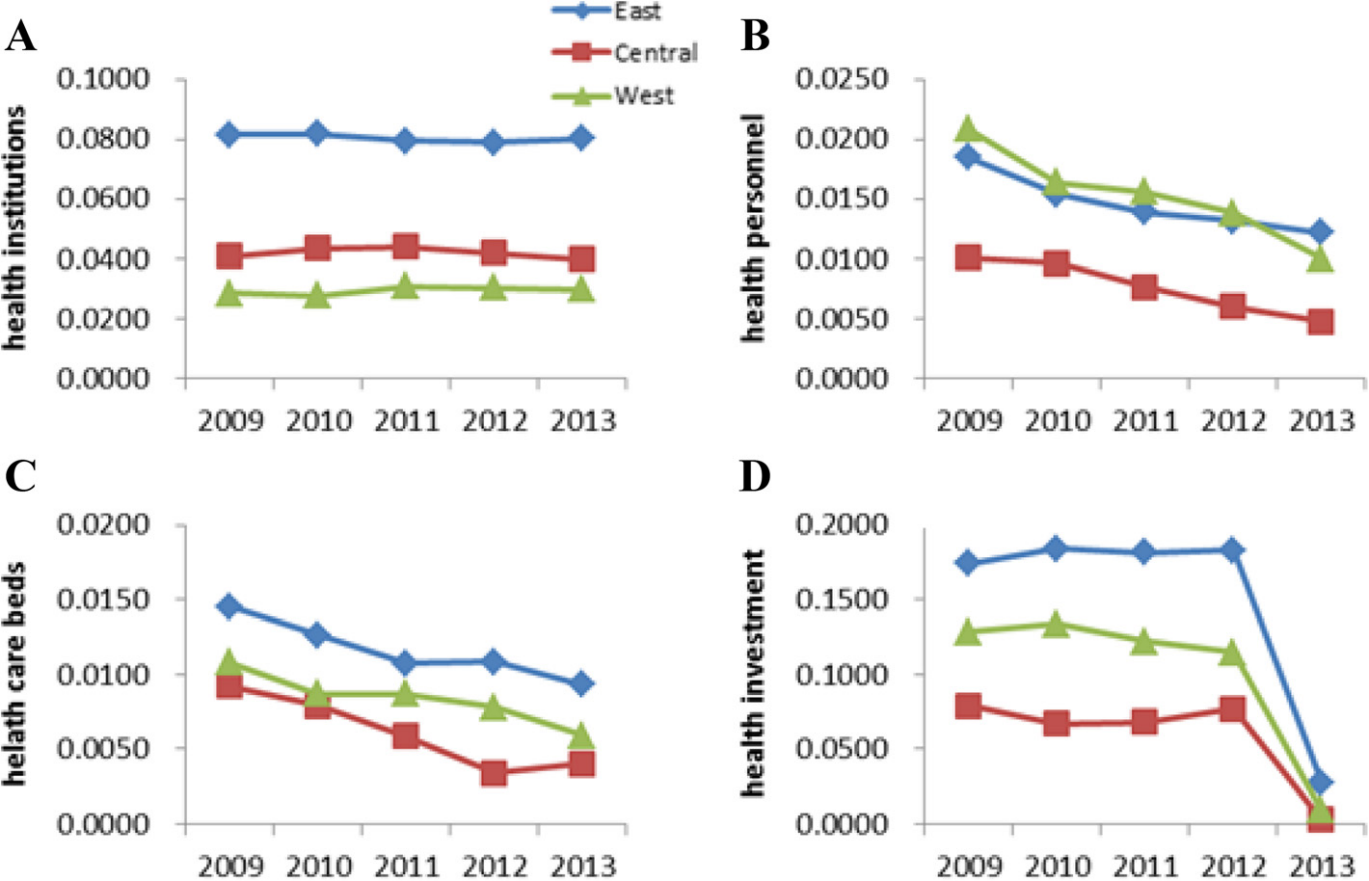
Theil index of health resource allocation from 2009 to 2013. **a**, **b**, **c** and **d** represents the four kinds of health resources. The figure shows the Theil index changes in the four categories of health resources. X-axis represents the year and Y-axis the Theil index value which was calculated by the formula (1) mentioned above. For details see text

The whole Theil index has been divided into 3 parts, eastern, central and western regions. The following Fig. 1 could be more intuitive to reflect the changes of Theil index during the year of 2009–2013. Theil index of the eastern region showed the highest except for the 2nd graph (health technical personnel). In terms of the health care institutions attribution, Theil index remained stable (about 0.08, 0.04 and 0.03 in eastern, central and western respectively) in the 5 years. Western region experienced the lowest extent of equity in health technical personnel distribution. But there was an obvious improvement in recent 5 years, Theil index in central region lower than that in eastern for the first time in 2013. Compared to the other 3 aspects, the improvement of fairness in health technical personnel allocation was the most obvious, with the values fell from 0.0185, 0.0101 and 0.0208 to 0.0122, 0.0048 and 0.0100 respectively in eastern, central and western regions during 2009–2013. For the health investment, the data showed a smooth change during the years of 2009–2013, while Theil index fell sharply into nearly 0 in the 3 regions.

### Theil index contribution rate of health care resources in different regions

**Fig. 2:**
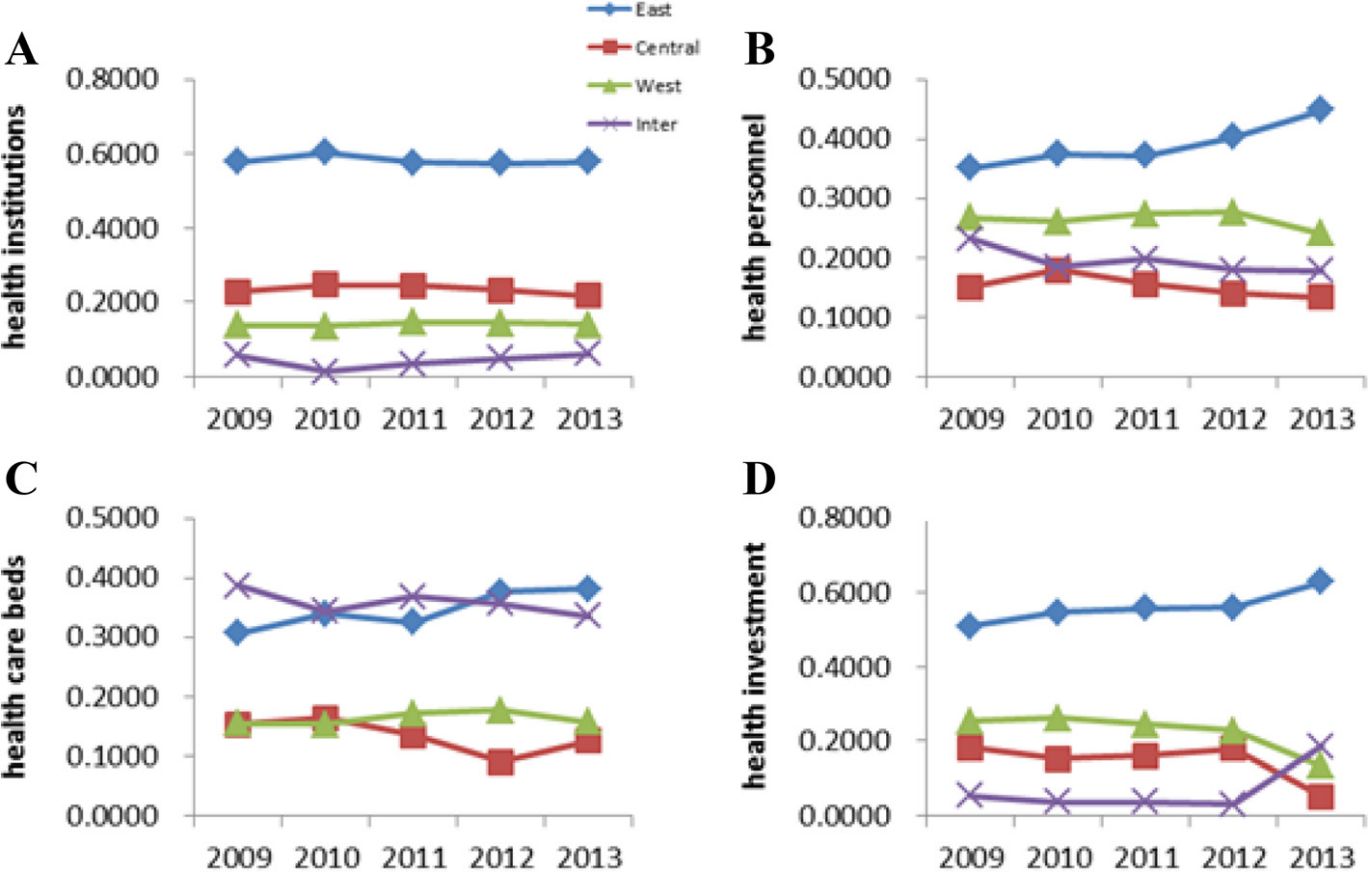
Contribution rate for Theil index of health resource allocation from 2009 to 2013 **a**, **b**, **c** and **d** represents the four kinds of health resources. The figure shows the contribution rate in the four categories of health resources which including the intra-regional part and the inter-regional part. The intra-regional part was divided into three regions which were mentioned in the text. X-axis represents the year and Y-axis the contribution rate for Theil index which was calculated by the formula (2), (3) and (4). For details see text

As shown in Fig. 2, the regional contribution rates were less than 10, 25, 40, and 20 % in respectively, lower than the proportion of inter-regional contribution rates. But in which, the gap between intra-regional and inter-regional in terms of beds in health care institutions attribute was smaller. The contribution rates were highest in eastern no matter calculated from which kind of resources. Among them, the contribution rate of institutional distribution and financial investment distribution in eastern even beyond 50 %. The overall trend of contribution rate in central and western region showed a steady decline, while the eastern was rising in recent years.

## Discussion

This study was a comprehensive nationwide study that assessed the trend of health resources allocation in China. The study found a steady rise in the total amount of health resources and per capita health resources ownership in China. The growth rate of total health resources was higher than that of population growth, which means that the opportunity to obtain the health services for national residents was increasing. The development of the social economy in China and the new round of medical reform program in 2009 may explain this phenomenon. Two of the key tasks of the reform were to improve primary health care service system and gradually realizing the equalization of basic public health services, which provided new opportunities for the development of health related causes. The first three years of the program, various regions make a deep development and improvement of their medical system according to the actual situation. Gradual implementations were made to improve the status of health resource allocation all around the country. Sun Zhigang, office director of health reform, summed up that the health reform in China had come into the "deep zone." Therefore we chose the data from 2009 to 2013 to conduct the trend analysis, which can reflect the impact of health reform on health resource allocation. From the results of this study, we can safely know that, the reform plan, a programmatic document of health development, has improved the overall fairness of the health resource allocation in China. However, further results for the distribution of health resources per 10,000 capita and per 10,000 km showed that there was a big gap among different provinces and regions. Meanwhile, there was a larger disparity in the geographic distribution of health resources than that in population distribution. Similar results also appeared in the study of other scholars [13, 14]. Implementations of supply-side-oriented resource allocation in China lead the limited concentrated in the densely populated and economically developed municipalities and developed areas, with an extreme shortage of health resources in less developed western regions which owned a vast and more dispersed population.

In order to make a thorough assessment of the distribution of health resources equity situation, we used an economic tool called Theil index, which can measure the "horizontal equity" of health resource allocation. The results showed a continuous improvement in the equity of health resource allocation in China. As the summary report 3 years after the new round of medical reform mentioned-China was trying her best to narrow the gap in health service between people at different levels from various regions, the implementation of regional health planning promoted a more reasonable inter-provincial health resource allocation. This situation was consistent with other studies [15, 16]. Equity in beds attribution was better than that in other 3 kinds of health resources, while related researches conducted by Zhang [17] and Huang [18] also found the lowest values of Theil index in beds. This result indicated that gap in the hardware health resources continued to decrease. For the aspect of health financial investment, the Theil index remained the highest from 2009 to 2012, which means the lowest fairness. From a practical point of view, this result is closely related to our health management system. Despite the unbalanced economic development situation or financial abilities in different provinces, the same health resource allocation standards should be abided. The areas with inadequate financial capacity could not implement the national standards, so the gap within regions might be bigger. At the same time, because of the loose management in marginal areas, the financial investment may not timely be decentralized to local level which aggravates problems of the inequity. The government in China was exploring the path of converting health surcharges levies such as environmental pollution tax into special fund of health care. For the allocation of institutions and health technical personnel, the situation was much better; we called it “relatively fair” [19]. But it was worth noting that Theil index values of health technical personnel showed the highest in western region, while the other 3 types of health resources allocation appeared highest in eastern. An earlier study in China also showed similar results [20]. This might be a very typical phenomenon in China. The economic situation in western region was relatively backward than the central region and eastern region. The number of less developed cities was larger than that in other areas. The capital cities have more opportunities to develop in comparison with ordinary cities in western. Health professionals prefer to serve in developed capital cities with better economic conditions, higher income and strong service abilities. This phenomenon of “capital city concentration” was more prominent in western region [21]. How to further guide the rational flow of health professionals was one of the key issues in health reform.

The contribution rate could help us to better understand the causes of inequity in health resource allocation. Results showed that the internal differences within regions contributed more to the inequity of health care allocation. The rate values were the highest in eastern region, which meant that the internal inequities in eastern were more serious. Gong’s study also found that the internal differences in the eastern region were the most important factor influencing the Theil index while the inter-regional differences in the contribution rate were small [22]. What must be concerned was the contribution rate of the eastern region which has been showing slow upward trend in recent years. The regions with best economic conditions undertook the worst conditions of equity in health resource allocation, which seemed a bit strange. But this phenomenon could be better explained if we consider it from the aspect of China’s national social conditions. The study found that, the amount of health resources in Beijing, Shanghai was much higher than that in Hebbel, Fujian which below the national average in eastern region. While the differences between the central or western interior provinces were relatively small. In fact, the differences of socio-economic development level in eastern region were significantly greater than other regions. Focusing only in the most developed provinces and cities, but ignoring the less develop areas or even sacrificing the interests of peripheral areas may result in the high degree of inequity in developed regions. In addition, another reason might be the more attention and investment to the western region or grassroots level in remote areas but ignoring the relatively backward provinces in eastern. The coastal areas and strong economic zones in eastern took advantages of their transportation or information to attract more investment for development also further widening the gap between regions in eastern. Therefore, the government should make a policy orientation to the less developed provinces in eastern when accelerating the health services development in central and western regions [23]. In short, the most reasonable allocation of health resources could be like the “equilateral triangle” [24]. It means that the flow of health resources should be based on the needs-oriented of population. But in fact, the health resource allocation model might be like “inverted triangular”. The Chinese government should act as a resource allocator to promote the improvement of the equity in health resource allocation.

Through this study, we can explore the current situation of equity in health resource allocation, and explore the problems which cause of the unfairness. A more targeted policy might be made via the analysis of the current weaknesses in China's health resources allocation. While the study also has some limitations; for one thing, the disaggregated data used in this manuscript can only reflect the health resource allocation status in the cut-off point of this work, with no completely reflection of the whole picture about it. Besides, Theil index cannot directly compare populations with different sizes as calculation is dependent on number of individuals in the population or group. For another, there might be other unmeasured factors influencing the differences observed, which need for further inquiry.

## Conclusion

The amount of health resources in China showed a state of steady increase from 2009 to 2013. Overall, the equity of health resource allocation improved gradually in these 5 years. The distribution of health investment was the most unfair when comparing with other 3 types of health resources, while the beds distribution appeared the most balanced. The disparities were mainly caused by the differences within the regions. The internal differences in the eastern region which made the highest contribution to the Theil index were relatively larger than other regions.

If the tough issues mentioned above could be solved effectively, China may become a good example for other countries with the same problems. Health resource allocation problem is a complex issue with a systemic and global, multiple disciplines of economics, sociology, political science and geography involved. The inequality phenomenon might be caused by various factors. We cannot make an accurate measurements and assessments of fairness via one kind of tools or several unilateral indexes. The theoretical depth of this study need to be strengthened and socioeconomic variables should be involved in future research.
